# Proteomic Biomarkers of Atherosclerosis

**DOI:** 10.4137/bmi.s488

**Published:** 2008-03-12

**Authors:** F. Vivanco, L.R. Padial, V.M. Darde, F. de la Cuesta, G. Alvarez-Llamas, Natacha Diaz-Prieto, M.G. Barderas

**Affiliations:** 1 Department of Immunology. Fundación Jiménez Díaz, Madrid, Spain; 2 Department of Biochemistry and Molecular Biology I, Universidad Complutense, Proteomic Unit, Madrid, Spain; 3 Department of Cardiology. Hospital Virgen de la Salud, SESCAM, Toledo, Spain; 4 Department of Vascular Pathophysiology. Hospital Nacional de Paraplejicos, SESCAM, Toledo, Spain

**Keywords:** atheroma plaque, atherosclerosis, biomarkers, proteomics, cardiovascular diseases

## Abstract

**Summary:**

Biomarkers provide a powerful approach to understanding the spectrum of cardiovascular diseases. They have application in screening, diagnostic, prognostication, prediction of recurrences and monitoring of therapy. The “omics” tool are becoming very useful in the development of new biomarkers in cardiovascular diseases. Among them, proteomics is especially fitted to look for new proteins in health and disease and is playing a significant role in the development of new diagnostic tools in cardiovascular diagnosis and prognosis. This review provides an overview of progress in applying proteomics to atherosclerosis. First, we describe novel proteins identified analysing atherosclerotic plaques directly. Careful analysis of proteins within the atherosclerotic vascular tissue can provide a repertoire of proteins involved in vascular remodelling and atherogenesis. Second, we discuss recent data concerning proteins secreted by atherosclerotic plaques. The definition of the atheroma plaque secretome resides in that proteins secreted by arteries can be very good candidates of novel biomarkers. Finally we describe proteins that have been differentially expressed (versus controls) by individual cells which constitute atheroma plaques (endothelial cells, vascular smooth muscle cells, macrophages and foam cells) as well as by circulating cells (monocytes, platelets) or novel biomarkers present in plasma.

## Introduction

Cardiovascular diseases are the leading cause of death and disability in developed countries. The diagnosis of most cardiovascular diseases is made on clinical grounds, but there are many ancillary techniques that are helpful in establishing or ruling out the suspected clinical diagnosis. Among these auxiliary techniques, biomarkers are getting an increasing and very important role.

Biomarkers provide a powerful approach to understanding the spectrum of cardiovascular disease. They have applications in several areas, such as screening, diagnosis, prognostication, prediction of recurrences, and monitoring of therapy. Advances in genomics, proteomics, metabolomics, and bioinformatics have revolutionized the search for numerous putative markers that may be informative with regard to the various stages of atherothrombosis.

Before they are used clinically, it is important to understand their specific indications, to standardized their analytical methods, to assess their performance characteristics and to establish the incremental value and cost-effectiveness of different markers for given clinical indications.^1^,^2^ Clinical application further requires the demonstration, in multiple prospective cohorts, that evaluation of the biomarker predictive of disease and adds to traditional risk factors.^3^

### Biomarkers

Generally, biomarkers are considered to be plasma measurements of molecules, proteins, or enzymes that provide independent diagnostic or prognostic value by reflecting an underlying disease state or condition.^3^ According to the definition proposed by the Medical Subject Heading (MeSH) a biomarker is a biological parameter that can be measured and quantified which serve as indices of health and physiology-related assessments, such as disease risks, environmental exposure and its effects, disease diagnosis, etc.^2^ An NIH working group standardized the definition of a biomarker in 2001, as “a characteristic that is objectively measured and evaluated as an indicator of normal biological processes, pathogenic processes, or pharmacologic responses to a therapeutic intervention.”^4^

The same NIH working group defined several types of biomarkers, as type O (a marker of the natural history of a disease and correlates longitudinally with known clinical indices; e.g. blood pressure in patients with systemic hypertension), type 1 (a marker that captures the effects of a therapeutic intervention in accordance with its mechanism of action; e.g. reduction in LDL- cholesterol with statins) and type 2 or surrogate end point (a marker that is intended to substitute for a clinical end point; a surrogate end point is expected to predict clinical benefit -or harm or lack of benefit or harm- on the basis of epidemiological, therapeutic, pathophysiological, or other scientific evidence; e.g. changes in carotid intima-media thickness as an index of atherosclerosis).^4^

A biomarker can be measured in a biological sample (blood, urine, etc), may be recorded in a patient (electrocardiogram, etc.) or can be an imaging test (echocardiogram, etc,). Biomarkers can indicate a variety of health or disease characteristics, including environmental factors, genetic susceptibility, clinical or preclinical disease, and accordingly can be classified as antecedent biomarkers (identifying the risk of developing an illness), screening biomarkers (screening for subclinical disease), diagnostic biomarkers (detecting overt disease), staging biomarkers (categorizing disease severity), or prognostic biomarkers (predicting future disease course, including recurrence and response to therapy, and monitoring efficacy of therapy). There is a significant overlap between these classifications of biomarkers, since type 0, 1 or 2 biomarkers can be used as antecedent, diagnostic or prognostic assessment of the patients depending on their clinical situation.

### The ideal biomarker

Independently of its use, the clinical value of a new biomarker will depend on its accuracy and reproducibility obtained in a standard fashion. Furthermore, it must be acceptable to the patient, easy to interpret by clinicians, must have high sensitivity and high specificity for the outcome it is expected to identify, and must be able to explain a reasonable proportion of the outcome independently of other established predictors. The information obtained by biomarker levels must have the potential to change clinical management of the patient in order to be cost-effective. The desired characteristics of a biomarker depend on its main use; diagnosis, prognosis, etc. Sensitivity is more important for diagnosis than for prognosis, for example.

### Definition of abnormal biomarkers values

The definition of the abnormal value of a biomarker is a key step before its use in clinical practice. There are several approaches to define the abnormal value of a biomarker: reference limit, discrimination limit or threshold defining risk.^2^ The reference limits are statistically derived cut-off points based on the distribution of values in a sample of reference; a sample of at least 200 people is needed to establish the reference range or the interval between the maximal and the minimum reference value.^5^ It must be known that a certain amount of normal patients can have abnormal levels of biomarkers defined statistically (for example, 5% of normal patients will be beyond the 95th percentile if this is the limit used). The reference interval can be moved up and down depending on the clinical significance of false-positive or false-negative results. The discrimination limit allows the separation of the distribution of patients with or without the disease evaluating the overlap existing between the two populations (with and without disease); the discrimination threshold can also be moved depending on the clinical significance of false-positive or false-negative results.^6^

And finally, the threshold defining risk identifies the level at which the risk increases steeply. For most cardiovascular risk factors, there is a continuum between the level of the risk factor and the presence of disease. Desirable levels are those that predict the lowest rate of disease, whereas the so-called treatment levels are the one in which current treatment have shown in randomized trials that can decrease the development of disease. While desirable level is an epidemiological deduction, the treatment level depends on the last tested treatments and usually goes down when more effective treatments are developed. High blood pressure^7^ and high cholesterol^8^ are two good examples of this distinction between optimal or desirable level and treatment levels.

### Evaluation of a biomarker

The precise evaluation of the performance of a biomarker depends on its main intended use. Biomarkers validation studies must perform a blind comparison of the new biomarker with a standard in a sample of consecutive patients with an appropriate spectrum of the disease.^9^ Two samples are usually needed, the derivation or training sample where the biomarker is firstly derived, and the validation or test set where the generalization of the preliminary results are tested.^10^ Usually, the performance of the biomarker is better in the first than in the second set which could be misleading. There are standards for designing and reporting studies of biomarkers validation in diagnosis and prognosis.

The accuracy of a biomarker is evaluated by estimation of sensitivity, specificity and likelihood ratios. The receiver operating characteristic (ROC) curves allow estimating the performance of a biomarker over a range of values. It illustrates the trade-off between sensitivity and specificity when a biomarker is used clinically for diagnosis.^11^ Likelihood ratios are calculated with sensitivity and specificity values and give the clinician more useful information: the likelihood of having a positive result in a diseased person (positive likelihood ratio) or of having a negative result in a healthy person (negative likelihood ratio).^12^ Biomarkers used to rule-out a dangerous disease must have a high sensitivity (small numbers of false negative tests and very low –<0,10- negative likelihood ratio) whereas when a biomarker is used to confirm a uncommon disease in asymptomatic people a high specificity is needed (small number of false positive tests and very high –> 10- positive likelihood ratio).^13^

A Bayesian approach, which includes pre-test probability of having the disease, is very important when using biomarkers to establish the presence of a disease in a given population.^14^ Furthermore, in order to be accepted as a routine screening test, a biomarker must demonstrate that a strategy of measuring it improves patient outcomes relative to a conventional strategy that does not include the given biomarker.^2^

Biomarkers are also evaluated in terms of their discrimination and calibration capabilities.^15^ Discrimination is the ability of a biomarker to separate disease from health (“cases” from “controls” in a cross-sectional study or the development of disease from the absence of it in a longitudinal study). The c statistic or concordance index is frequently used for this purpose as a metric of overall performance of a test; it is equivalent to “the area under the curve” in the ROC curve and expresses the probability than in two randomly paired individuals (with and without disease) the biomarker can properly tell apart the one with disease. On the other hand, calibration analyses the relationship between the ability of a biomarker to predict risk with the actual risk of subgroups of patients, and it is particularly important when the question is the numeric probability of disease in a given patient. The Hosmer-Lemeshow goodness-of-fit statistic, which divides the sample into deciles of risk and compares the observed with the expected number of events, is often used as an indicator of model calibration.^16^ Using this methodology estimation of risk can be performed in individuals using score systems that include the biomarker, such as in the Framingham cardiovascular risk score.

It is important to evaluate the incremental value of a new biomarker demonstrating the elevated risk of an outcome associated with higher levels of the new biomarker after adjustment for other established risk factors. Hazard ratios (relative risk estimates from a Cox model) are frequently used for this purpose, as well as a probability value test of the significance of the biomarker in the multivariable models. Nevertheless, the relative added values of new biomarkers is best evaluated by estimating the increment to the c statistic compared with that from a model that incorporates other previously known predictors.^17^ For example, the Framingham coronary heart disease score is composed of several biomarkers and has a c statistic of 0.74–0.76, which is considered appropriated^18^; this c statistic is not incremented adding other biomarkers such as C-reactive protein that have been shown to be associated with cardiovascular disease. In spite of that, some biomarkers such as homocysteine are useful for specific populations of patients even if they do not increment the c statistic in the total population.^19^

In studies using genetic biomarkers there is a major concern about false-positive associations with disease (or phenotypes) resulting from numerous additional factors,^20^ and a replication of results in multiple independent samples is essential.^21^

Obviously, the analytical method must be standardized to obtain accurate results of the biomarkers. Good quality control within the laboratories is needed.^22^

There are many biomarkers for cardiovascular disease,^23^–^25^ with different levels of evidence in relationship with diagnosis, prognosis and pathophysiologic development of the disease.^2^,^3^,^26^ Jaffe et al. have analysed the current situation of biomarkers for acute cardiac disease and have concluded that there are established biomarkers (troponins), potentially outdated markers (CK-MB, myoglobin and CK isoforms), emerging markers (C-reactive protein, B-type natriuretic peptide) and developing markers (sCD40 ligand, myeloperoxidase, ischemia-modified albumin, pregnancy-associated plasma protein-A, choline, placental growth factor, cystatin C and fatty acid binding protein). Some emerging and developing markers will help provide a better framework for treatment of cardiovascular diseases in the future.

### Discovery of new biomarkers

The discovery of new biomarkers is a very challenging process especially in cardiovascular disease where many genes and pathophysiologic pathways are involved. Two potential complementary approaches can be applied: deductive or inductive strategies. In the deductive method (knowledge based) a direct understanding of the biological processes underlying atherosclerosis is needed to search for new molecules that can be used as markers, whereas in the inductive method (unbiased approach) the study of many molecules with the use of current techniques to characterize the bio-molecular profile of a stage of the disease. The “omics” tools are becoming very useful in the development of new biomarkers in cardiovascular disease. Among them, proteomic is especially fitted to look for new proteins in health and disease and is playing a significant role in the development of new diagnostic tools in cardiovascular diagnosis and prognosis.^27^

## Proteomic Approach for the Study of Cardiovascular Diseases

Proteomics is the large study of the structure and function of proteins; it includes the rapidly evolving field of clinical proteomics, which aims to identify proteins involved in human disease and understand how their expression, structure and function cause illness. Proteomics-based screening test would compare the proteins expressed in blood or a tissue sample or cells with protein patterns from patients known to have a particular disease. This technology has identified proteins that offer promise as diagnostic or prognostic markers, or as therapeutic targets in a range of illness, including vascular diseases.

### Proteomics in the field of atherosclerosis

The mechanism by which atherosclerosis develops is still unclear, but some clear factors related to this development are hypercholesterolemia and accumulation of inflammatory cells in the vascular wall. The first signal is the formation of fatty streaks under the endothelium of arteries. The recruitment of monocytes to the lesion leads to an inflammatory response and cytokine secretion. This fact induces the proliferation of vascular smooth muscle cells (VSMC) from the media and subsequent neointima formation. At the same time, necrosis of VSMCs and macrophages contributes to the formation of a fibrous cap that covers a lipid-rich core. This cap keeps stable until the expression of proteases promotes the degradation of extracellular matrix and rupture of the plaque, inducing thrombus formation.^28^–^30^ Several recent reviews on vascular proteomics have been reported.^31^–^34^

Until recently, the usual approach has been to study the role of a candidate protein supposedly involved in the formation or progression of the atherosclerotic lesion. However, with the appearance of new proteomic techniques of protein separation (2-DE, MudPIT), and their identification by mass spectrometry (MS), the evaluation of thousands of proteins at once is now possible. At present, it is possible to perform a differential proteomic approach on a variety of biological samples, including cells, tissues or biological fluids. In the context of biomarker discovery, biological fluids such as plasma or urine, represent the most logical compartment for investigation because of their easy access. However, the analysis of plasma is challenging because it contains a plethora of proteins resulting from a transient complex metabolic state of the whole body.^35^ Atherosclerosis is characterized by focal plaques from which proteins can diffuse in limited amounts into plasma where their detection and subsequent identification is difficult as they are masked by the large quantities of major plasma proteins such as albumin or immunoglobulins. Recent technological improvements in depletion of abundant plasma proteins compatible with proteomic analysis should facilitate discovery of biomarkers of vascular origin, directly related to the pathology.^36^,^37^ Another approach consists of the analysis of either vascular cells under pathological conditions or atherosclerotic plaques, as compared to healthy cells or tissues. A choice must be made either to study cellular/tissue proteins or secreted/released proteins, the latter being more likely to be found in plasma.

### Proteomic analysis of the atheroma plaque

The study of human atherosclerotic lesions (usually carotid or coronary artery segments bearing atheroma plaques) is complicated owing to their cellular heterogeneity (endothelial cells, VSMC, macrophages and foam cells, leukocyte, erythrocytes, platelets) and the different degree of development and complication. Proteins are the major mediators of most pathological processes and are the most suitable molecules for use as biomarker and risk factors, as well as targets for disease treatment. Thus, to have a better understanding of atherosclerosis progression it is necessary to identify and characterize the expressed proteins in the arterial wall and in the atheroma plaques. Most likely, the proteins expressed (and/or secreted) for the multiple cell types present in the atherosclerotic plaque, can potentially contribute to the pathogenesis of atherosclerosis. In fact many proteins (growth factors, lipid associated proteins, membrane receptors, tissue enzymes, etc.) have all been implicated in the complex process of atherogenesis. Thus, profiling of protein expression from atherosclerotic tissue samples can provide a molecular snapshot of the functional and pathological state of the plaques. With these goals in mind several experimental approaches can be applied: 1) to determine all the expressed proteins solubilizing the tissue and analyzing the proteins by 2-DE or MudPIT.^38^–^40^ 2) to focus in a selected group of proteins using antibody arrays or even restrict the analysis to a very specific set such as those involved in signaling pathways using reverse phase arrays and specific antibodies (phosphoproteome).^41^ 3) to study the proteome of arteries and atherosclerotic plaques using direct tissue proteomics (DTP), a new methodology which allows the identification of proteins directly in formalin fixed paraffin-embebed tissue samples.^42^ 4) by MS-Imaging, a suitable method to obtain protein and peptide profiles and images (two-dimensional protein maps) from thin frozen tissue sections.^43^ 5) to focus on particular subproteomes (intima, extracellular proteins and proteoglycans, neovascularization associated proteins, etc.)^44^ and 6) to asses the proteins expressed in relatively pure cell populations, obtained from the atherosclerotic sample tissues, using laser micro-dissection.^45^ Several examples of these approaches are described below:

1. When 2-DE profiles of human stable lesions plaques where compared with plaques containing a thrombus^38^ these showed 71 spots exclusive from stable plaques and 29 exclusive from plaques containing a thrombus. The most interesting finding is the difference found in the expression of α_1_-antitrypsin (ATT) between the stable plaque and the one containing a thrombus. The stable plaque showed the expression of 6 isoforms of ATT versus only one isoform present in the thrombotic plaque, so the up regulation of ATT could be considered as a protective strategy against atherosclerosis. Using an apoliprotein-E (apo-E) deficient mice model, the protein changes analyzed during various stages of atherogenesis were studied by 2DE and MS.^39^ An average of 1500 spots were visualized in the 2DE aorta sample from which 79 protein were found to be altered during the different stages of atherogenesis.^39^ Likewise in a rat model of coronary atherosclerosis induced by a high cholesterol diet, 46 proteins were differentially expressed in the aortic tissue of atherosclerotic rats in comparison with aortas from control animals. Among the upregulated proteins were redox enzymes and those with decreased levels included HSP-27, calcium calmodulin kinase II inhibitory protein and fructose biphosphate aldolase.^40^
2. Using antibody arrays, Slevin et al.^41^ have identified novel proteins associated with the development of unstable human carotid plaques. They compared protein expression in carotid endarterectomy samples histologically defined as stable and unstable. Their results could indicated that the modulation of these novel cell signaling proteins can be useful in therapy of angiogenesis and apoptosis, designed to reduced unstable plaque formation.^41^
3. using DTP in 35 human coronary atherosclerotic samples more than 800 proteins were identified, including extracellular matrix proteins, lipid binding and metabolism associated proteins, inflammation related proteins and apoptotic-cell phagocytic ligands and receptors. The presence of these proteins in the tissue was confirmed by immunohistochemistry.^42^
4. MS-imaging of tissues is a powerful tool for visualizing the spatial distribution of proteins on the surface of the tissue and it has been applied in several pathologies.^43^ However it has not been applied yet on atherosclerotic or normal arteries for a number of reasons. In contrast, the application of TOF-SIMS in the vascular pathology area has permitted to generate, for the first time, molecularions images of human atherosclerotic plaques and rat aortic walls.^46^,^47^ In TOF-SIMS imaging detailed chemical information of the upper most molecular layers of a solid surface can be obtained at a sub-micrometer lateral resolution.^48^ All the molecules with mw <1500 Da can be, in principle, detected. Surface rastering from atherosclerotic lesion thin-sections generated numerous secondary ion signals including nonesterified fatty acids, cholesterol, vitamin E, phosphatidic acids, phosphatidylinositols and triglycerides. Specific patterns for most of these compounds could be observed on apparently homogeneous tissue as it is the vascular muscle cell of the intima.^46^
5. In humans proteoglycans of intimal hyperplasia are believed to play an important role in the development of atherosclerosis. A comparison between atherosclerotic-prone internal carotid artery and the atherosclerotic-resistant internal thoracic artery showed an enhanced deposition of lumican in atherosclerotic-prone arteries.^44^ This significant difference is present before the atherome plaque formation, but the factors responsible for this difference are unknown. Another proteomic approach that analyzed a particular subproteome was performed by Martinet et al.^49^,^50^ by Western array using 823 monoclonal antibodies. They compared human atherosclerotic carotid arteries versus healthy mammary arteries and reported seven proteins with enhanced expression levels of 5-fold relative difference. One of them, ALG-2 is a positive mediator of apoptosis, phenomenon that is believed to be implicated with plaque instability. The diminished levels observed for ALG-2 suggest a survival mechanism of the atherosclerotic plaque.
6. The isolation of macrophage foam cells from arterial lesions has been applied to study the gene expression of these cells.^45^ This method is now been used for proteomic analysis of foam and endothelial cells from atherosclerotic lesions (De la Cuesta et al. unpublished results).

### Proteomic analysis of the secretome of atheroma plaque

An alternative strategy to the study of the tissue is to examine the proteins secreted by atherosclerotic plaques. The interest of the definition of the atheroma plaque secretome resides in that proteins secreted or released by the artery wall or the cells within the tissue, constitute the best candidates for potential novel biomarkers that could be detected subsequently in blood or plasma. In order to easily identify those proteins which are secreted or released by atherosclerotic plaques in extracellular compartments, we have described^51^–^53^ a novel approach by which secretomes from normal and atherosclerotic arteries can be obtained incubating endarterectomy samples in a protein-free culture medium and analyzing the proteins secreted into the medium. By focusing on only the secreted proteins found in the cell culture media, there would be an intended bias toward those proteins that would have a higher probability of later being found in plasma. We have shown that, in human carotid atherosclerotic plaques the more complex the lesion, the higher the number of proteins secreted by the plaque.^51^ Among the differentially secreted proteins decreased levels of phosphorylated HSP-27 were found in the culture medium of atherosclerotic plaques in comparison with healthy mammary arteries. These data were supported by the finding that HSP-27 plasma levels were diminished in patients with carotid atherosclerosis compared to normal controls indicating that this protein could be a novel biomarker of atherosclerosis in relation with healthy subjects.^54^ The pathological significance of decreased HSP-27 plasma levels is unknown but it is conceivable that the decreased HSP-27 released by atherosclerotic plaques could be caused by proteolysis of the protein in the tissue owing to the action of the proteases present in the plaques.^55^ A potential drawback in the study of secretomes using cultures of artery segments is that many of the proteins identified in the culture supernatants can be of plasma origin (contaminants). Thus, to validate this approach it is essential to identify the true secretome, in other words, the identification of proteins which are actively synthesized and secreted by the normal and pathological arterial wall. Very recently we have studied the effect of atorvastatine alone or combined with amlodipine in the protein secretion profile of atherosclerotic plaques.^52^ It is well known that a combined treatment produces a higher reduction in the incidence of cardiovascular events than each drug administered alone. The addition of both drugs to complicated atherosclerotic segments normalized the levels of the majority of the released proteins. Thus, the application of this proteomic strategy has permitted the identification of novel therapeutic targets by which these drugs could promote their additive effects.^52^

All the cell types that make up the atheroma plaque are able to secrete proteins, thus contributing to the composition of the secretome. For example, in the secretome of VSMCs in culture,^56^,^57^ 18 different proteins have been identified, with a wide range of biological functions. Similarly, the secretome of human macrophages has been recently reported.^58^

### Proteomic analysis of individual constituents of atheroma plaque

An alternative to studying blood vessel is to use cultured cells under conditions mimicking the situation in atherosclerotic lesions. This is an advantageous system that permit the selection of cell type, treatment with different agents, stimulants and drugs and to determine the effects on the expressed proteins.

#### Endothelial cells (EC)

The formation, development, progression and complication of atherome lesions are closely related to endothelial dysfunction. Hence the study of EC is of prime importance. Endothelial cells can be harvested from a variety of vessels but most of our knowledge in vascular pathology come from HUVECS (human umbilical vein endothelial cells).^59^ The initial studies described the protein expression map of primary cultures of HUVECS and several proteins were identified implicated in apoptosis, senescence and coagulation, including cathepsin B, a protease upregulated in atherosclerotic lesions with senescence-associated phenotypes.^60^ The effect of oxLDL on EC function is one of the earliest events in the pathogenesis of atherosclerosis. The effect of oxLDL on EC was studied by Kinumi et al. (2005)^61^ that found three proteins, nucleohosmin, stathamin and nucleolin, that were differentially expressed and phosphorylated. It was suggested that the process of phosphorylation/dephosphorylation of these proteins contributed to the suppression of EC proliferation. The effect of genistein (a component of soy) on the expression of proteins in EC exposed to oxLDL reversed 27 out of 47 proteins altered by oxLDL. These data help to explain some of the proatherogenic properties of oxLDL and the capacity of genistein to protect from these effects.^62^ Using bovine aortic endothelial cells, a significant number of proteins were identified with altered levels in response to shear stress, a well known factor contributing to atherogenesis. Some of these proteins are involved in signaling pathways including integrins and G-protein coupled receptors.^63^ The caveolae and lipid rafts associated proteins of EC have been described recently and could serve as the basis for future studies considering the key role of these membrane microdomains in signal transduction, cellular transport and cholesterol homeostasis.^64^,^65^

Two annotated 2-DE protein maps of EC are available on the web at (http://www.huvec.com; http://www.pharma.ethz.ch/bmm/div/HUVEC/HUVEC/.)

#### Vascular smooth muscle cells (VSMC)

Cellular proliferation and migration of VSMC are considered to be key events in the pathogenesis of atherosclerosis. This is feasible because VSMC are cells capable to switch between a contractile and an activated phenotype under the effect of soluble serum factors.^66^ As a consequence of endothelial dysfunction vascular permeability increases and many serum components come into direct contact with VSMC inducing the change from a resting VSMC to an activated-proliferating phenotype. To identify changes in specific proteins associated with both cellular phenotypes, a comparative 2-DE analysis was performed on protein extracts from quiescent rat aorta VSMC and VSCM exposed to growth factors. More than 100 proteins were upregulated and 154 downregulated in activated cells compared with the quiescent ones.^67^ Likewise, proteins associated with either hyperplastic or hyperthrophic growth due to the exposure of VSMC to growth factor were determined. Among the upregulated proteins there were chaperones, factors involved in protein synthesis and cytoskeletal proteins such as vimentin and actin.^68^ The effect in the protein expression levels induced by treatment of human aortic VSMC with oxLDL versus nLDL was studied using antibody arrays. Among the upregulated proteins were adhesion molecules, signal transduction proteins and proteins involved in lipoprotein metabolism.^69^ Underexpressed proteins included cytoskeleton proteins and growth factors. Lee et al. (2006)^70^ studied alteration in protein expression in VSMC under oxidative stress (exposing the cells to H_2_O_2_), a phenomenon that plays a key role in the pathophysiology of vascular diseases. The most relevant VSMC proteins altered were those related to antioxidant, cytoskeleton and muscle contraction.^70^

As mechanical forces play an important role in the pathogenesis of atherosclerosis the expression changes in the protein profile due to hemodynamical stress were also studied in VSMCs. This study showed that three proteins, gelsolin, HSP-27 and CapZ had their levels altered.^71^

#### Macrophages and foam cells

Inflammation plays a central role throughout the entire atherosclerotic process and monocytes/macrophages (and infiltrating T lymphocytes) are the main cells involved in this inflammatory reaction. Different studies have reported the proteome of monocytes in basal and under proinflammatory conditions.^72^–^75^ In atherosclerotic lesions monocytes migrate into the vessel wall and differentiate into macrophages which become lipid-loaded after uptake of oxLDL. Thus, several studies examined the effect of oxLDL on the protein expression levels of macrophages.^76^–^79^ Collectively it was shown that more than 100 proteins were altered as consequence of the oxLDL treatment and they can be grouped in metabolic proteins, components of differentiation pathways and antioxidant proteins. A group of antioxidant enzymes were also over-expressed by mature dendritic cells which are present in advanced atherosclerotic plaques. The potential role as biomarkers of these proteins expressed by macrophages is at present unknown.

### Circulating cells

The proteome of the majority of circulating cells has recently been described including erythrocytes, neutrophils, lymphocytes, monocytes and platelets.^31^,^32^ Herein only monocytes and platelets will be discussed as they are providing new proteins that could potentially be considered as biomarkers.

#### Monocytes

To search for novel biomarkers in circulating monocytes, using proteomic approaches can be a fruitful strategy considering that monocytes are easily accessible and essential cells that participate throughout the atherosclerotic process. However it is important to be aware of two aspects: first is the heterogeneity of human monocytes as determined by the expression of surface markers (CD14, CD16, MHC class II, CD32, CCR5/CCR2) that allows to classify monocytes in several subsets which reflect development stages and specialization within their environments.^80^ In addition it is essential to consider the proteome variance in the control population when performing protein profiling comparisons of monocytes derived from disease versus control populations.^81^ The role of monocytes in the progression of the atheroma plaque has been described above. It has been recently shown that a population of monocytes/macrophages emigrate from atherosclerotic lesions and re-entry into the blood. Thus, monocytes may serve as reporters, providing information reflecting directly the state of the atherosclerotic process.^82^ A good method to retrieve this information from monocytes is to analyze their expressed proteins. Hence we have examined whether circulating monocytes from acute coronary syndrome (ACS) patients express specific proteins that could serve as individual markers or define a characteristic profile. It was shown that 21 proteins were differentially expressed by monocytes from ACS patients in comparison with healthy subjects.^83^ The number of proteins with altered expression in ACS was maximal on admission, and decreased progressively, until the expression of only five proteins was abnormal at six months. These five proteins were identically altered in stable patients. Thus, six months after an ACS, the acute changes in monocyte protein expression disappear, showing a pattern similar to that of stable CAD. Among the identified proteins, a 32 kDa protein corresponding to the mature form of Cathepsin D was shown to be upregulated in monocytes of ACS patients. Interestingly, treatment with atorvastatin could modulate some of the altered proteins in the monocytes from ACS patients. Treatment with atorvastatin favored the reversion to normal levels in 8 protein, 2 proteins were partially normalized, and 11 proteins appeared for the first time as consequence of the statin treatment.^84^

#### Platelets

Platelets play a critical role in hemostasis of the vessel wall: when the endothelium is damaged, activated platelets adhere to the ECM components contributing to the healing process. The proteome of platelets in a basal state has been extensively studied and numerous proteins involved in essential cellular functions, such as signal transduction, modulation of the cytoskeleton and apoptosis have been identified.^85^–^90^ However the proteome of platelets is particularly dynamic, depending of their state of activation. Thus, the study of activated platelets is very important in relation with the atherosclerotic process since activated platelets adhere to atherosclerotic lesions and could be a source of plaque biomarker as they release proteins after activation. In thrombin activated platelets more than 300 proteins have been identified in several studies.^91^–^97^ Some of the identified proteins were not previously attributed to platelets (secretogranin, cyclofilin A, calumenin), were not present in normal arteries but were clearly identified in human atherosclerotic lesions. Thus, these proteins are very promising candidates as novel biomarkers.

### Plasma

Human plasma is a rich source of proteins and other metabolites which reflect the physiological or clinical status of patients. The protein concentration in plasma is tightly controlled, thus variations in concentration can be considered as an indicator of the current state of health.^98^ Hence plasma is a primary clinical specimen with 20 major proteins comprising 99% of the plasma proteome and the rest of the proteins making up 1% of the plasma content. The challenge is thus to reach this 1% where it is supposed some novel biomarkers can be potentially found.^99^ For this purpose it is essential to deplete plasma of the most abundant proteins.^100^,^101^ To identify low-abundance proteins, plasma samples from 53 patients (a total volume of 6 liters) with coronary artery disease (CAD) and 53 control subjects (6 liters of plasma) were pooled and analyzed by LC-MS/MS, after removal of albumin and immunoglobulins and enrichment in smaller proteins (<20–40 kDa). Of the 731 proteins identified, 95 were differentially expressed in CAD patients and could be candidates for validation studies in larger clinical studies.^102^ Very recently it has been shown that proteins derived from tissues can be detected in plasma directly by MS analysis, providing a conceptual basis for plasma protein biomarker discovery and analysis.^103^ One of the major clinical complications of atherosclerosis is the impairment of blood flow to the lower extremity which is commonly referred to as peripheral artery disease (PAD). For a number of reasons PAD is underdiagnosed^104^ despite this disorder affects more than 10 million individual in USA and is also prevalent in Europe and Asia. Very recently Wilson et al.^105^ have studied the proteomic profile of 371 subjects in three sequential groups (discovery, initial validation and validation) and found that plasma levels of β2-microglobulin are elevated in patients with PAD and that β2-microglobulin levels independently correlate with the severity of the disease. These are very promising data although some potential limitations have been discussed.^104^ Similarly, the MALDI-TOF spectra of peptides from the sera of normal and patients with myocardial infarction (MI) produced patterns that could help in the diagnosis of MI.^106^ Kierman et al.^107^ have used a multiplexed MS immunoassay (MSIA) for the detection of MI and two novel biomarkers, serum amyloid A1α and S-sulfated transthyretin, were found to be present in the sera of these patients. Both proteins were subsequently validated in a large cohort of patients. In mice it has been tested whether protein microarray based measurements of circulating proteins can predict the severity of atherosclerotic disease. From a total of thirty inflammatory markers a subset of proteins were identify that classify and predict severity of atherosclerotic disease.^108^

Lipoproteins are shuttling hydrophobic microparticles, formed for lipids and proteins, that travel from sites of absorption and production to sites of storage and excretion trough the blood system. Lipoproteins are involved in atherogenesis but the molecular mechanisms by which participate in the genesis and progression of atherosclerotic plaques are not fully elucidated. In recent years different proteomic approaches have been used to analyze the protein composition of HDL, LDL and VLDL and numerous novel proteins (and isoforms) have been identified in the three types of lipoproteins.^109^–^115^ Thus further research is needed to establish their putative role in atherosclerosis and their potential usefulness as biomarkers.

The HUPO Plasma Proteome consortium has identified a group of 345 cardiovascular-related proteins that could constitute a baseline proteomic blueprint for the future development of biosignatures for cardiovascular diseases.^116^ These proteins can be organized in eight groups: markers of inflammation, and/or cardiovascular disease, vascular and coagulation, signaling, growth and differentiation, cytoskeletal, transcription factors, channel/receptors and heart failure and remodeling. Likewise, Anderson, L. (2005) has compiled a list of 177 candidate biomarker proteins with reported associations to cardiovascular disease and stroke.^117^ It is reasonable to consider that all these proteins may serve as the basis for further research. However this approach is inherently limiting and fails to allow for discovery of totally novel proteins involved in cardiovascular diseases. It is more challenging to use unbiased proteomic approaches to identify undiscovered novel proteins that can be true biomarkers and potential targets for the treatment of these pathologies.

### Urine

Urinary proteomics is emerging as a powerful non-invasive tool for diagnosis and monitoring a variety of human diseases. Urine presents a rich source of information related to function of many integral organs.^118^ Changes in the pattern of urinary protein excretion are not necessarily restricted to nephrourological diseases. Thus, microalbuminuria is a clinically important marker not only of early diabetic nephropathy but also of concomitant cardiovascular disease. In this sense, Zimmerli et al.^119^ have tested whether signature of urinary polypeptides can contribute to the existing biomarkers for coronary artery disease (CAD). They examined a total of 359 urine samples from 87 patients with severe CAD and 283 controls using capillary electrophoresis on line to ESI-TOF-MS, characterizing more than 100 polypeptides. They found a panel of 15 polypeptides which distinguished between presence and absence of disease and identified five of them which corresponded to collagen type I or type III fragments. These collagens are predominant proteins in the arterial walls and are present in the thickened intimae of atherosclerotic lesions.

## Abbreviations

MeSH: Medical Subject Heading
ROC: Receiver Operating Characteristic
CK-MB: Creatine-Kinase Myocardial Band
VSMC: Vascular smooth muscle cells
MudPIT: Multidimensional Protein Identification Technique
2-DE: Two-dimensional electrophoresis
MS: Mass spectrometry
DTP: Direct tissue proteomics
ATT: α1-anti-tripsyn
SIMS-TOF: Secondary ion mass spectrometry time of flight
HSP: heat shock protein
EC: Endothelial cells
HUVECS: Human umbilical vein endothelial cells
oxLDL: oxidized low density lipoprotein
ACS: Acute coronary syndrome
CAD: Coronary artery disease
PAD: Peripheral artery disease
MALDI-TOF: Matrix assisted desorption ionisation time of flight
MI: Myocardial infarction
MSIA: Multiplexed mass spectrometry immunoassay
HUPO: Human Proteome Organization
